# Wireless, Skin-Mountable EMG Sensor for Human–Machine Interface Application

**DOI:** 10.3390/mi10120879

**Published:** 2019-12-14

**Authors:** Min-Su Song, Sung-Gu Kang, Kyu-Tae Lee, Jeonghyun Kim

**Affiliations:** 1 Department of Electronics Convergence Engineering, Kwangwoon University, Seoul 01899, Korea; 2 Department of Physics, Inha University, Incheon 22212, Korea

**Keywords:** electromyogram, human–machine interface (HMI), optimization, biosignal, wireless, wearable

## Abstract

The development of advanced technologies for wireless data collection and the analysis of quantitative data, with application to a human–machine interface (HMI), is of growing interest. In particular, various wearable devices related to HMIs are being developed. These devices require a customization process that considers the physical characteristics of each individual, such as mounting positions of electrodes, muscle masses, and so forth. Here, the authors report device and calculation concepts for flexible platforms that can measure electrical signals changed through electromyography (EMG). This soft, flexible, and lightweight EMG sensor can be attached to curved surfaces such as the forearm, biceps, back, legs, etc., and optimized biosignals can be obtained continuously through post-processing. In addition to the measurement of EMG signals, the application of the HMI has stable performance and high accuracy of more than 95%, as confirmed by 50 trials per case. The result of this study shows the possibility of application to various fields such as entertainment, the military, robotics, and healthcare in the future.

## 1. Introduction

Recently, as the integration technology of semiconductors developed, devices and sensors decreased in size, enabling various novel applications, including wearable sensors [1,2,3,4]. In particular, non-invasive wearable sensors collecting electrical signals from electrodes attached to the skin are used as key tools for obtaining various biosignals, such as electrocardiography (ECG) [5,6], surface electromyography (sEMG) [7], electromyography (EMG) [8,9], electroencephalography (EEG) [10], and so forth. These sensors are used in the medical field for the diagnosis of disease states or the monitoring of health conditions. For example, ECG sensors can be used to diagnose cardiomyopathy [11] and angina pectoris [12], and EEG sensors can be used to diagnose schizophrenia [13]. In particular, EMG sensors measuring the electrical signals of muscle movements are used not only for the diagnosis of diseases but also for the rehabilitation of prosthetic patients [14,15] or human–machine interface (HMI) applications such as robots and drones [7,16,17]. 

Various wearable EMG sensors were researched and developed, but there are some limitations for HMI applications such as their bulky size, wires from external equipment, external power supply, etc. These limitations apply not only to EMG sensors but also wearable sensors in general. Several all-in-one sensors using (near field communication) NFC and Bluetooth were recently developed to overcome these problems [6,18,19]. Additionally, since the muscle mass varies from person to person and the EMG signal can be different for each electrode attachment position, the calibration and post-processing of data are necessary to adjust signal variance [20,21,22]. Therefore, for a more practical HMI application, it is necessary to study all-in-one devices that can be fully wireless and stand-alone in terms of data processing without any connection to an external system.

Here, we developed an integrated EMG sensor that consists of electrodes, a sensor, and Bluetooth low energy (BLE), which can address the challenges described above. Removing the wires from the external equipment and fabricating it in a skin-attachable form led to the improved portability of the EMG sensor. This sensor can optimize bioelectrical signals by programming a microcontroller unit (MCU) to minimize signal variances from different persons and electrode mounting locations. In the classification process required for HMI application, the signal range of the contraction and relaxation states is set properly, which can assist users when exploiting the sensor without modifying circuits and calibrating data. Additionally, a moving average filter is used to increase accuracy, in terms of obtaining the signal, converting it to a specific value, and sending it to the receiver to control the target. In this process, BLE is employed for wireless communication between the transmitter and receiver. The EMG sensor described in this paper is expected to be useful in terms of self-diagnosis, health monitoring and in various fields such as entertainment [23], the military, and robotics [24,25], as well as giving limbless patients the ability to control the target through various gestures [25].

## 2. Materials and Methods

### 2.1. Architecture of the Electromyography (EMG) Sensor for Human–Machine Interface (HMI) Application

**Figure 1:**
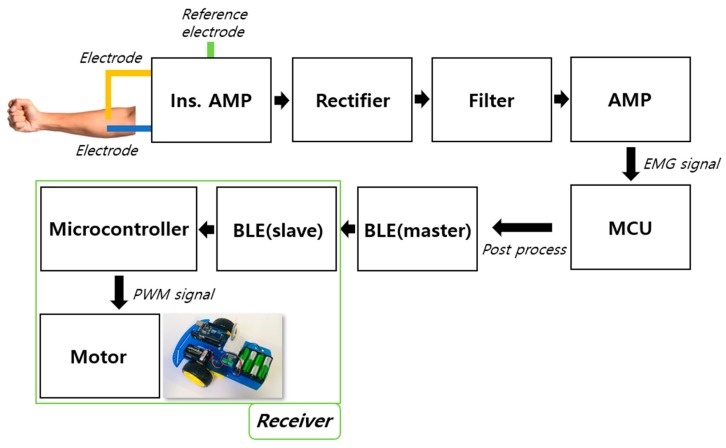
Block diagram of the electromyography (EMG) sensor.

Figure 1 shows a block diagram of the EMG sensor system. The raw EMG signal is obtained through the instrumentation amplifier and the three electrodes; two of them are placed on the skin in the measured muscle area, and one electrode is placed on the skin in a less muscular area, which is used as the ground point. The raw signal and EMG signal can be found in Figure S1 (Supplementary Materials). The MCU’s analog-to-digital converter (ADC) is used for data collection, and a full-wave rectification is used to convert the alternating current (AC) component of the raw signal into a direct current (DC) component. The rectified signal is amplified and smoothed; then, the collected EMG signal is read via the MCU and sent to the receiver through universal asynchronous receiver–transmitter (UART) communication using the BLE module, which enables wireless communication. The receiver is operated and controlled using the EMG signal.

### 2.2. EMG Sensor and Wearable Design

**Figure 2:**
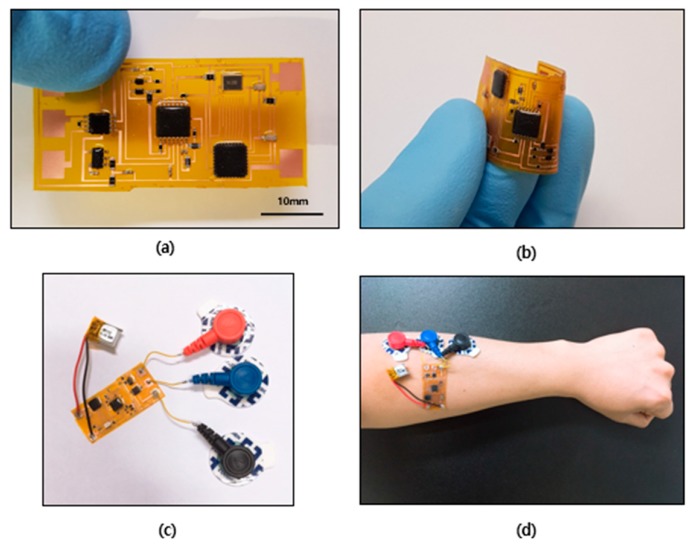
(**a**) Image of the EMG sensor. Scale bar, 10 mm. (**b**) Image of the bent EMG sensor. (**c**) Image of the EMG sensor with battery and electrodes. (**d**) Image of the EMG sensor attached to the forearm.

The image of the EMG sensor is shown in Figure 2a. The sensor is 40 mm × 20 mm, and the size of the sensor can be reduced by varying the layout as need. It consists of integrated circuit (IC) chips that are commercial off-the-shelf surface-mounted devices (SMDs), which are light and small in size. These components were fabricated on a copper sheet with laminated polyimide (PI). This flexible printed circuit board (FPCB) has the advantage of being able to bend, making it suitable for human skin, which is soft and curved. Also, a silicon-based elastomer is used in the process of packaging, which helps the sensor to be flexible (Figure 2b). In other words, this sensor is suitable for wearable devices that can be attached to human skin. The electrodes are attached to the three pads on the left part of the sensor, and the power is connected to the two pads on the right part of the sensor. Photographs of the sensor with the battery and electrodes can be found in Figure 2c,d. In this study, commercial electrodes were used; however, the use of epidermal electrodes is expected to improve the wearability of the sensor.

This EMG sensor is connected to a BLE module via a flexible flat cable (FFC) (0.5mm A-10P 150 mm), as shown in Figure S2 (Supplementary Materials).

## 3. Experiments

### 3.1. Circuit Design and Configuration

**Figure A1:**
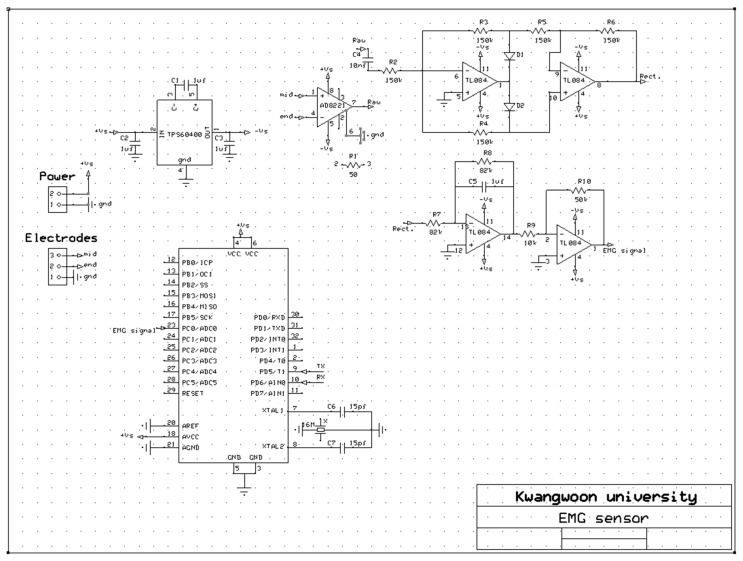
Schematic of EMG sensor.

The raw EMG signal is collected with the instrumentation amplifier AD8221 (Analog Devices, Norwood, MA, USA) and three electrodes (H124SG, Covidien, Dublin, Ireland). Two electrodes are attached to muscle areas, while the reference electrode is attached to a less muscular area. The obtained raw EMG signal is full-wave rectified by two diodes 1N4148 (Vishay, Malvern, PA, USA) and a quad amplifier TL084. The rectified signal is transferred to the MCU after smoothing and amplifying. For smoothing and amplifying, the amplifier TL084 (Texas Instrument, Dallas, TX, USA) was used. Figure A1 (Appendix A) shows the whole circuit. This design was modified and carried out according to a previous study [26].

### 3.2. Design Wireless Communication System and MCU

BLE module HM10s and MCU atmega328p were used in this study. They exchange data via UART communication. The BLE module of the transmitter was set as the master, and the module of the receiver was set as the slave. To use the MCU, the boot loader has to be burned. Serial peripheral interface (SPI) communication is used between the computer and the MCU to avoid any overlap with UART communication.

### 3.3. Fabrication of the EMG Sensor and BLE Module

To fabricate the EMG sensor, a layer of photoresist (AZ4620) was spin-coated on the copper sheet (18um; SME co., Yongin, Korea) laminated with PI for 30 s at 3000 rpm. Then, it was annealed on a hot plate (C-MAG HS 7; IKA, Staufen, Germany) at 110 °C for 120 s. For photolithography, we used a mask aligner (cooluv-100; Jaesung Engineering, Ansan, Korea) for 45 s. AZ300 MIF and CE-100 were used for development and wet etching, respectively. Development took about 90 s, and etching took about 270 s. We soldered the IC chip on the fabricated FPCB using solder paste (52In 48Sn; Indium co., Clinton, NY, USA) A soldering iron (WD1; Weller, Besigheim, Germany) and a gun (6966KO; Weller, Besigheim, Germany) were used in the soldering process. Lastly, we used the PDMS mixed at a 10:1 ratio of 184 silicone elastomer base and 184 silicone elastomer curing for packaging. The BLE module was similarly packaged by PDMS mixed at a 10:1 ratio of 184 silicone elastomer base and 184 silicone elastomer curing, and it was connected to the EMG sensor through an FFC (0.5 mm A-10P 150 mm).

### 3.4. Classification of EMG Signals

To confirm that the raw EMG signal waveform changes with muscle movement, an oscilloscope (DSO-X-2012A; Agilent technology, Santa Clara, CA, USA) was used. The rectified signal is measured by a digital multimeter (TDM2065; TESTLINK, Gwangmyeong, Korea). An atmega328p ADC is used to record the EMG signals by reading the voltage with a division ratio of 0 to 1023. This was scaled up to 0 to 5000 via programming. We measured the voltage according to a specific gesture and used that as a boundary. Then, we wrote a conditional statement according to the boundary for optimization to control the target.

### 3.5. HMI Application

A remote-controlled (RC) car (DV1802-KIT; Entriex Co., Ltd., Seoul, Korea) was used to test the performance of the sensor. Four 1.5 V alkaline batteries were used to power the motor, and 3.3 V was transferred via a microcontroller to the BLE module of the receiver. The circuit of the RC car was configured to operate the motor. The speed was set using a pulse width modulation (PWM) digital output; when the RC car was still, it stopped, the palm pinned forward, and the fist was reversed. The RC car is set to operate when a certain signal came in for each gesture. The experiments involved a volunteer (male, 26 years old) seated on a chair.

## 4. Results and Discussions

**Figure 3:**
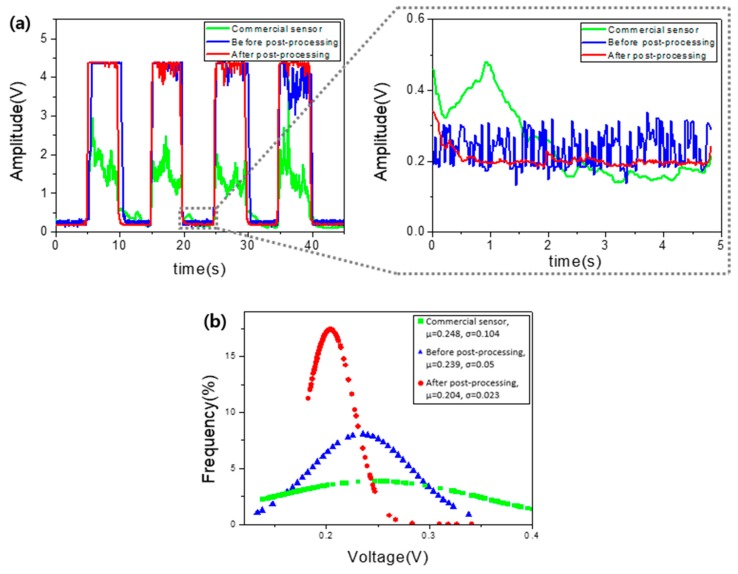
(**a**) Monitored EMG signals using the EMG sensor and commercial sensor. (**b**) Standard normal distribution graph related to stability.

This soft, flexible, lightweight EMG sensor can be attached to the skin to receive electrical signals according to physical motions, and the collected data are sent to the receiver via BLE. It is driven at voltages between 3.3 V and 5 V. A 5-V power supply was used in the process of classification of data. A total of three electrodes were used, two electrodes attached to the forearm and a reference electrode attached to the elbow. The mounting position of the electrodes for the corresponding data can be found in Figure S3 (Supplementary Materials). The EMG signal recorded by the MCU’s ADC can be seen in Figure 3a, which shows a comparison between a commercial sensor and our sensor. The data were measured with a squeezed fist, which was released at five-second intervals. The results show that our EMG sensor had higher gain than the commercial sensor and showed more stable data. An enlarged view of the data collected from 20 to 25 s can be seen to the right of Figure 3a, and the standard normalization of these data can be seen in Figure 3b, where µ is the average of the collected data and σ is the standard deviation. The EMG sensor’s standard deviation was 0.023, and the commercial sensor’s standard deviation was 0.104, which means that the EMG sensor output a more stable value.

The maximum output voltage when using muscles is proportional to the supply voltage of the amplifiers because they fall into the saturation region. Using these characteristics, conditional statements were set based on the bounding values for each pose. The signal range of contraction and relaxation states was minimized, and the range of the intermediate stage was maximized. In order to obtain precise data, abnormally acquired data for a short time are ignored and a moving average filter is used with 200 data. This filter is expressed as the formula shown below.

(1) $$\bar{x_{n}}=\frac{x_{n-k+1}+x_{n-k+2}\;+\;\ldots +x_{n}}{k},$$

where k is the number of data. Equation (1) can also be expressed in a different way as follows:

(2) $$\bar{x_{n-1}}=\frac{x_{n-k}+x_{n-k+1}\;+\;\ldots +x_{n-1}}{k}.$$

Subtracting Equation (2) from Equation (1), we can obtain the following recurrence relationship:

(3) $$\bar{x_{n}}=\bar{x_{n-1}}+\frac{x_{n}+x_{n-k}}{k}.$$

**Figure 4:**
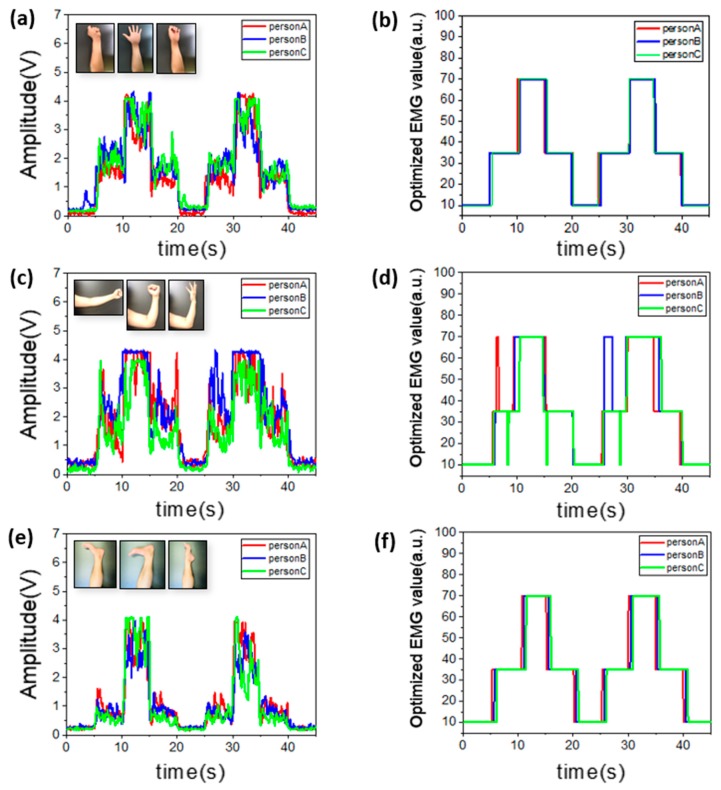
EMG signal sensing according to attachment site and different persons. (**a**) EMG signal on the forearm with three types of gestures. (**b**) Optimized EMG signal on the forearm. (**c**) EMG signal on the biceps with three types of gestures. (**d**) Optimized EMG signal on the biceps. (**e**) EMG signal on the calf with three types of gestures. (**f**) Optimized EMG signal on the calf.

In this way, this sensor can be used to acquire high-quality and optimized EMG signals on the muscular regions of the body. Figure 4 shows examples of signals obtained from the forearm, biceps, and calf muscles. Data were recorded through three gestures for each site. Figure 4a shows the first demonstration that proceeded in the forearm, which involved staying still, clapping hands, and clenching fists. The two electrodes were located in the forearm, and the ground electrode was located on the elbow, i.e., a less muscular area. Figure 4b shows an example of the optimized EMG value scaled from 0 to 100. As can be seen in the graph, there was no fluctuation of the value; thus, the correct value could be obtained upon data reception. Figure 4c shows the signal from the biceps. In general, the biceps muscle volume is greater than that of the forearm; thus, the EMG signals are larger than measured in other parts. In the biceps, it is difficult to classify three postures; thus, the hand was rotated outward to give more strength. Compared with values at other sites, there were more errors because the muscle mass is higher than in other parts, which makes it difficult to control, as shown in Figure 4d. The accuracy can be improved with additional training and experience or by attaching electrodes to areas with less muscle like the side of the arm. Figure 4e shows the signal from the calf. In this experiment, the ground electrode was attached to the shin with less muscle. Since the calf muscles of the volunteers for the experiment did not have a lot of muscle mass, the signal of Figure 4e was smaller compared to other parts. Nevertheless, it was confirmed that the boundary value was clear according to each gesture, and the error was eliminated when optimization was performed. The optimized data from the calf is shown in Figure 4f. In addition, it is possible to obtain EMG signals in a similar way from the thighs, abs, lats, etc. As a result, this sensor is able to obtain an optimized signal regardless of the attachment area or muscle size, and more accurate information is expected upon applying deep learning methods [27]. The mounting position of the electrodes for the corresponding data can be found in Figure S3 (Supplementary Materials).

**Figure 5:**
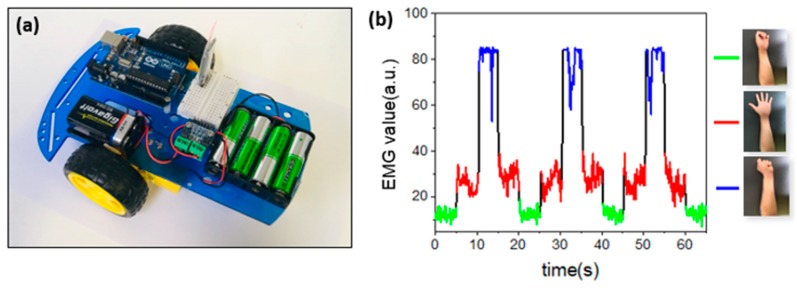
Human–machine interface (HMI) and EMG signals. (**a**) Image of a remote-operated RC car. (**b**) EMG signals recorded on forearms associated with three gestures.

The versatility in terms of location and the easy optimization in these experiments suggest many possibilities in health monitoring, as well as in HMI technologies based on EMG. The measurement of EMG signals on the forearms demonstrates the latter possibility through the control of the RC car (Figure 5a). Firstly, the BLE module on the sensor was set as the master, and the receiver was set as the slave; they were automatically paired through a prior setting. The MCU and BLE module communicated via UART to exchange data, and the EMG signal data were transmitted to the BLE module by the MCU, and finally sent to the BLE module on the receiver side. Since the microcontroller and BLE module on the receiver side also communicated via UART, they could exchange data. As a result, the microcontroller processed the collected data and allowed the RC car to operate under certain gestures. The rotational speed of the motor was adjusted by pulse width modulation (PWM) from the microcontroller. The information about the RC car can be seen in Figure S4 (Supplementary Materials). As shown in the figure, the three gestures illustrated in Figure 5b corresponded to distinct commands, i.e., stop, forward, and backward. 

**Figure 6:**
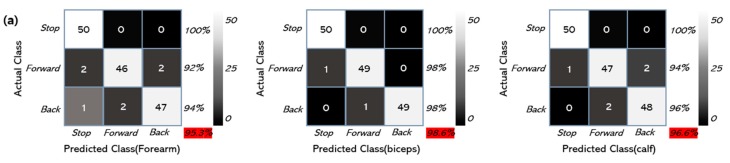

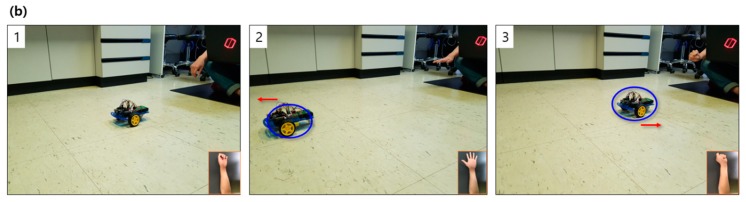
(**a**) Confusion matrix that describes the performance of the classification test. (**b**) Image of the RC car controlled by EMG signals from the forearm. Insets show the control gestures.

The RC car was configured to operate upon the receipt of a specific value for each gesture, with the results shown in Figure 6a. As an example, the success rate in the forearm for the “backward” command was 94% for 50 trials. The overall accuracy of all three classifications was 95.3%. The accuracy of the “stop” command was 100%, and that of the “forward” command was 92%. The overall accuracy of all three classifications in the biceps and calf was 98.6% and 96.6%, respectively. These results are different from the result in Figure 4, as the biceps were easy to maintain in the same muscle condition. For similar reasons, even in the same posture, it was difficult to reach 100% accuracy because it was hard to maintain the same muscle state. The accuracy can be improved with additional training and experience, enhanced classification algorithms, and the use of additional/different features in the data. The results of experiments using different sensors can be seen in Figure S5 (Supplementary Materials), and a comparison can be seen in Table 1. As demonstrated in Figure 6b, the movement of the RC car could be successfully controlled in this manner. Video S1 (Supplementary Materials) shows the movement of the RC car from another angle. Additional classification can be obtained by further dividing the range according to gesture or by using more electrodes. If the receiver has another BLE module that can pair with the module we used, it is expected to be used not only in RC cars but also in various fields such as drones, robots, and so forth.

## 5. Conclusions

Our research aimed to develop an EMG sensor that can be used by different persons with different amounts of muscle, grafting it to the HMI. This sensor is flexible, soft, light, and small; thus, it is suitable for human skin as a wearable form. It can be used as a system with a design that includes a sensor, MCU, and Bluetooth. Also, the sensor’s potability was improved through removing the wires from external equipment, thus enhancing wearability. This EMG sensor solves the problem of EMG signals changing when the user and the attachment positions of the electrode are varied through post-processing. Wireless communication through Bluetooth allows the control of distant targets as desired. In this study, the performance was confirmed by controlling an RC car, and the possibility of the HMI was confirmed with an accuracy of 95% or more. For the healthcare field, it could be used to self-monitor and strengthen muscle conditions. We hope that the results of this study will be used in a variety of fields to improve the quality of human life, especially for limbless patients through wireless control.

## Figures and Tables

**Table 1:** Comparison with other sensors. EMG—electromyography.

Sensor Type	EMG Sensor	Untreated Sensor	Commercial Sensor
Mean (V)	0.204	0.239	0.248
Standard deviation (V)	0.023	0.05	0.104
Accuracy (%)	95.3	92.7	85.3

## Supplementary Materials

The following are available online at https://www.mdpi.com/2072-666X/10/12/879/s1: Figure S1. The raw EMG signals associated with clenching the fist; Figure S2. (**a**) Image of the connected BLE module. (**b**) Image of the edge of FFC cable. (**c**) Area where FFC cable is connected to EMG sensor; Figure S3. (**a**) Mounting position of electrodes in Figure 3, Figure 4a, Figure 5 and Figure 6. (**b**) Mounting position of electrodes in Figure 4c. (**c**) Mounting position of electrodes in Figure 4e; Figure S4. (**a**) The front view of the RC car. (**b**) The rear view of the RC car; Figure S5. (**a**) Confusion matrix that describes the performance using the commercial sensor. (**b**) Confusion matrix that describes the performance using the untreated sensor; Video S1. EMG Test.
