# Supplementary material for: Wireless, Skin-Mountable EMG Sensor for Human–Machine Interface Application
Source: Micromachines (Basel). 2019 Dec 14;10(12):879. doi: 10.3390/mi10120879 (PMC6952934; doi:10.3390/mi10120879)
Supplement: Supplementary file 1 [file micromachines-10-00879-s001.zip › micromachines-652200-supplementary.pdf]

Supporting Information: Wireless, Skin-Mountable EMG Sensor for Human-Machine Interface Application

Min-Su Song [1], Sung-Gu Kang [1], Kyu-Tae Lee [2,*] and Jeonghyun Kim [1,*]

[1] Department of Electronics Convergence Engineering, Kwangwoon University, Seoul 01899, Korea;

[2] Department of Physics, Inha University, Incheon 22212, Korea;

* Correspondence: ktlee@inha.ac.kr (K.-T.L.); jkim@kw.ac.kr (J.K.) ; Tel.: +82-32-860-7653 (K.-T.L.); +82-2-940-5554 (J.K.)

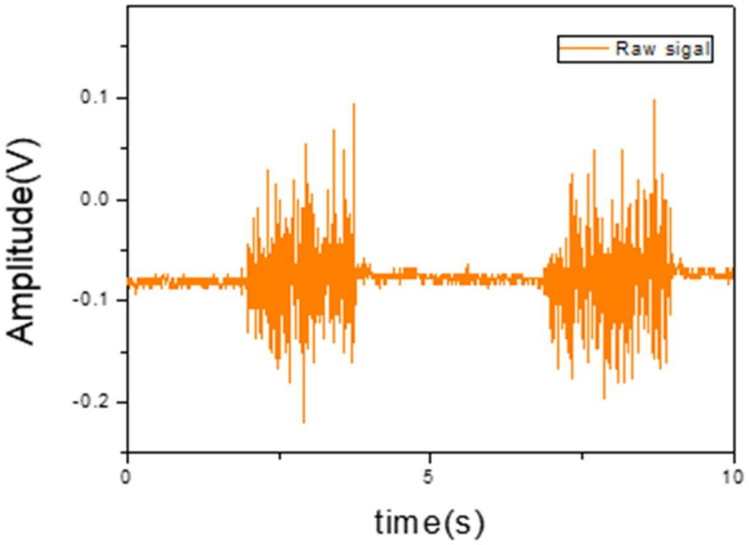

Figure S1. The raw EMG signals associated with clenching the fist.

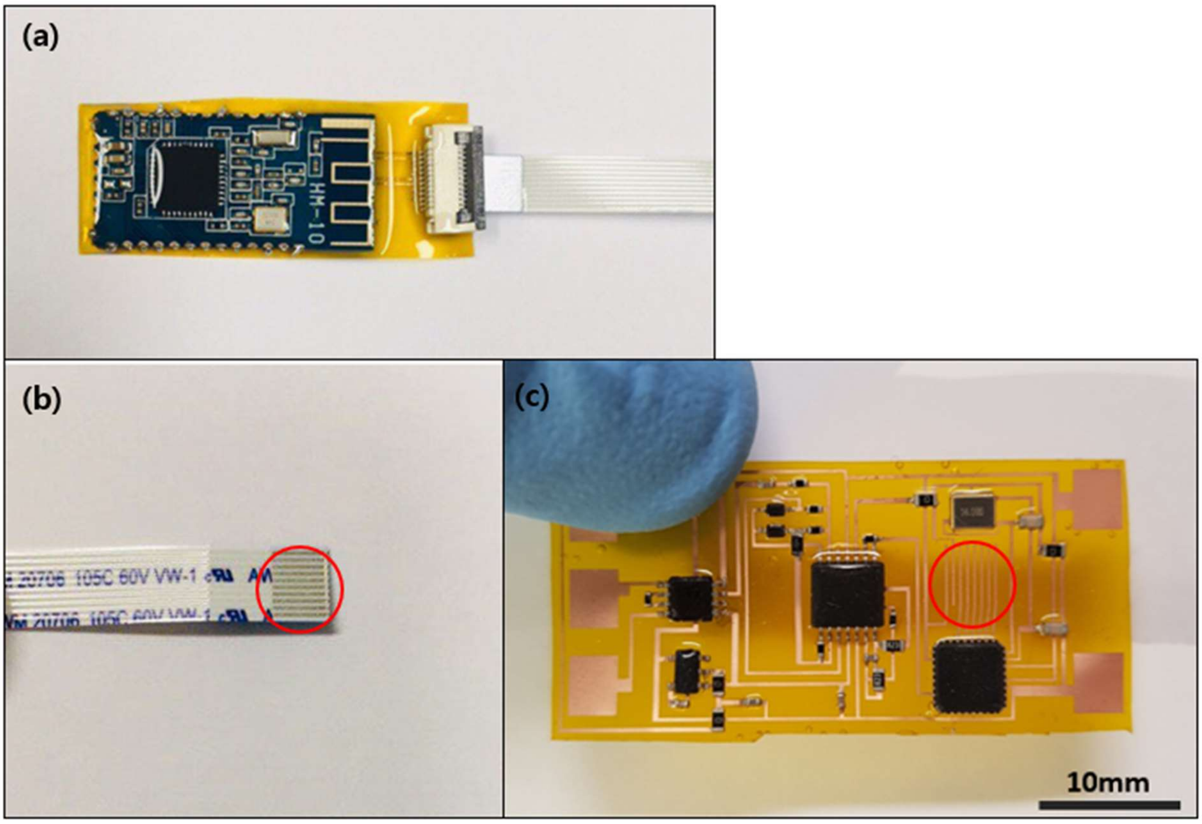

Figure S2. (**a**) Image of the connected BLE module. (**b**) Image of the edge of FFC cable. (**c**) Area that FFC cable is connected in EMG sensor.

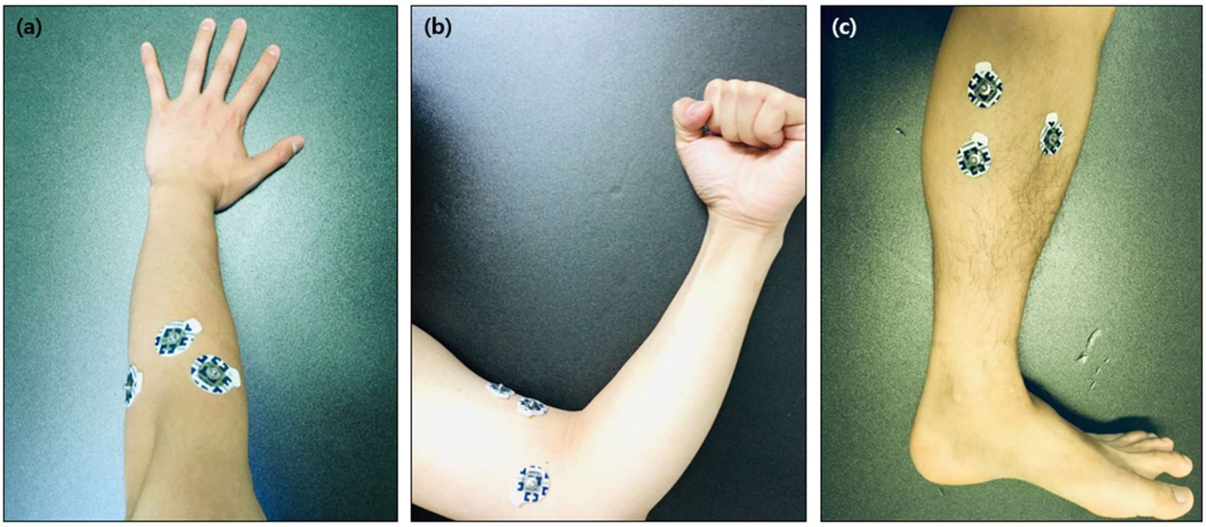

Figure S3. (**a**) Mounting position of electrodes in Figures 3, 4a, 5 and 6. (**b**) Mounting position of electrodes in Figure 4c. (**c**) Mounting position of electrodes in Figure 4e.

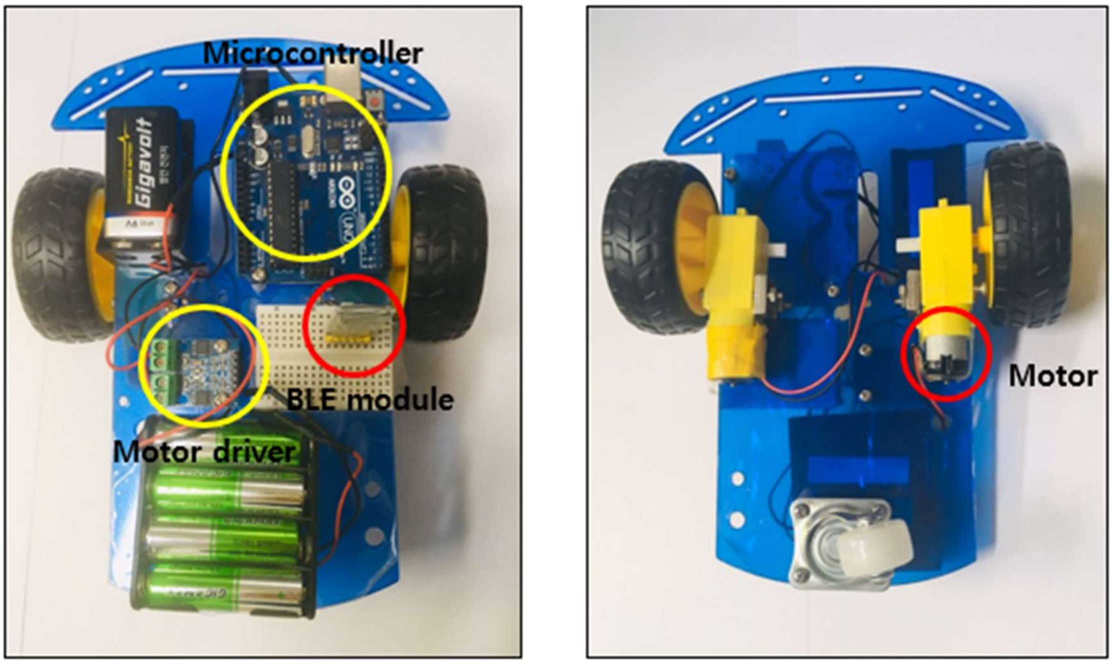

Figure S4. (**a**) The front view of the RC car. (**b**) The rear view of the RC car.

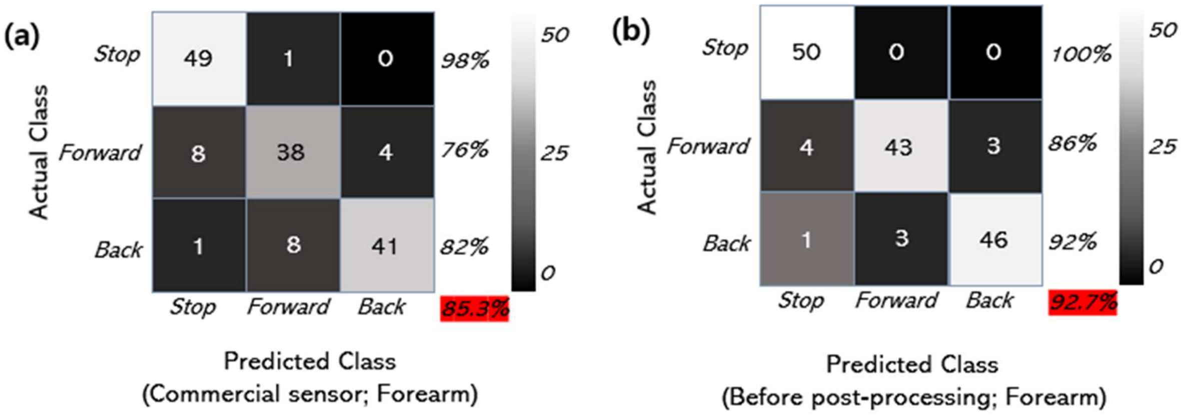

Figure S5. (**a**) Confusion matrix that describes the performance using commercial sensor. (**b**) Confusion matrix that describes the performance using untreated sensor.
